# Prolonged Glycation of Hen Egg White Lysozyme Generates Non Amyloidal Structures

**DOI:** 10.1371/journal.pone.0074336

**Published:** 2013-09-16

**Authors:** Sudeshna Ghosh, Nitin Kumar Pandey, Atanu Singha Roy, Debi Ranjan Tripathy, Amit Kumar Dinda, Swagata Dasgupta

**Affiliations:** Department of Chemistry, Indian Institute of Technology Kharagpur, Kharagpur, India; Aligarh Muslim University, India

## Abstract

Glycation causes severe damage to protein structure that could lead to amyloid formation in special cases. Here in this report, we have shown for the first time that hen egg white lysozyme (HEWL) does not undergo amyloid formation even after prolonged glycation in the presence of D-glucose, D-fructose and D-ribose. Cross-linked oligomers were formed in all the cases and ribose was found to be the most potent among the three sugars. Ribose mediated oligomers, however, exhibit Thioflavin T binding properties although microscopic images clearly show amorphous and globular morphology of the aggregates. Our study demonstrates that the structural damage of hen egg white lysozyme due to glycation generates unstructured aggregates.

## Introduction

Non-enzymatic glycation of proteins is a post-translational modification process which involves covalent bond formation between free amino groups of proteins and reducing sugars [1]. It results in the modification of proteins which leads to the formation of groups of heterogeneous compounds commonly referred to as advanced glycation end products (AGEs) [2,3]. AGEs play a crucial role in aging processes and debilitating diseases such as Alzheimer’s disease [4]. Conformational alterations in the protein structure result due to glycation, which affects the physical and functional properties of proteins [5]. Glycation can also lead to protein aggregation [4]. Formation of toxic protein aggregates commonly known as amyloid fibrils, is associated with neurological disorders such as Alzheimer’s, transmissible spongiform encephalopathies etc [6,7]. AGE modified amyloid fibrils have been found in the brain tissues of patients suffering from Alzheimer’s, transmissible spongiform encephalopathy and the islets of Langerhans of diabetic patients [8-10]. Fibrils possess a common core crossed β-sheet structural motif [11] and glycation facilitates the formation of the cross-linked beta structure [4]. Moreover, a recent report has shown that the glycated Aβ_1-42_ peptide with an altered secondary structure is more toxic with respect to the pathogenesis associated with Alzheimer’s disease [12]. These findings have encouraged researchers to investigate the structural changes of proteins that evolve due to glycation and to explore the possible link between glycation of proteins and amyloid formation.

Hen egg white lysozyme (HEWL) is known to possess a well-defined three dimensional structure, folding mechanism and thermodynamic parameters [13-16]. Human lysozyme is a structural homologue of HEWL, which is known to be involved in a systemic amyloidosis disease due to a point mutation in the lysozyme gene [17]. Fibrils obtained from human lysozyme *in vitro* were found to be similar with those obtained from patients and also fibrils obtained from human lysozyme bear a notable resemblance with HEWL fibrils [18,19]. A recent report has shown that human lysozyme fibrils can induce the secretion of innate immune receptors where the cross β-sheet structure plays a crucial role [20]. Membrane activity and the ability of inducing apoptosis in neuroblastoma cells by HEWL fibrils support the use of HEWL as a suitable model to study the mechanistic aspects of amyloid formation *in vitro* [21,22].

The potential glycation sites in HEWL are considered to be the N-terminal α-amino group, ε-amino group of lysine residues and guanidino group of arginine residues [23]. Reports are available on glycation of HEWL [24-29]. HEWL was found to bind with AGEs and exhibit a protective character due to its crucial role in the elimination of AGEs generated *in vivo* [30]. Several studies have revealed the involvement of different proteins in the formation of AGEs and their relation with protein aggregation and amyloid formation [4,31-38]. Based on the information above and considering the role of lysozyme in our natural immune system [^3^9, 40], we have chosen HEWL to examine the effect of glycation and its consequences on HEWL aggregation *in vitro*. In the present article, we have treated HEWL over a prolonged period (~180 days) in the presence of D-glucose (glucose), D-fructose (fructose) and D-ribose (ribose) at pH 7.4 at 37 °C. Glycation of HEWL was characterized using different spectroscopic and microscopic techniques. HEWL was found to form cross-linked oligomeric species in the presence of all the three sugars. Ribose was found to exert the most proficient role in comparison to fructose and glucose. Our study demonstrates that glycation of HEWL promotes aggregation but no fibrillar species was observed. Recent studies have shown that methylglyoxal (glycating agent) favors formation of native like aggregates of insulin [41] and cytochrome c [42]. This study will be beneficial in terms of understanding the effects of glycation on the structural aspects of proteins and its consequences in protein aggregation.

## Materials and Methods

### Materials

Hen Egg White Lysozyme (HEWL), Thioflavin T (ThT) were purchased from Sigma Chemical Co. (St. Louis, USA) and used as received. D-glucose, D-fructose and D-ribose and all other chemicals were obtained from SRL (India). Protein molecular weight marker (14.3-97.4 kDa) was purchased from Bangalore, Genei.

### Preparation of glycated HEWL: prolonged incubation to facilitate aggregation

HEWL (10 mg/ml) [ε_280_=37646 M^-1^cm^-1^] [43] was incubated in 0.1 M sodium phosphate buffer of pH 7.4 at 37 °C for 30 days in the absence and presence of three reducing sugars such as D-glucose (glucose), D-fructose (fructose) and D-ribose (ribose) of 0.5 M concentration in each case. This incubation was extended up to 180 days to promote aggregation. Sodium azide (1 mM) was used to prevent bacterial growth. Incubation process was carried out under sterile conditions. Aliquots were withdrawn at definite time intervals and sodium phosphate buffer (10 mM) of pH 7.4 was used for dilution to achieve the final desired concentration of protein in each study. In each study, the control is native HEWL incubated in the absence of sugars at pH 7.4 at 37 °C keeping the other conditions similar to that of sets (HEWL-glucose/ HEWL-fructose/ HEWL-ribose).

### Fluorescence measurements: steady state fluorescence

The formation of AGEs was monitored using different fluorescence spectroscopic techniques. At definite intervals (in days), aliquots were withdrawn and used for fluorescence measurements using a final protein concentration of 5 µM. Fluorescence of the samples was monitored using excitation wavelengths of 295, 335, 350 and 370 nm respectively in a Horiba Jobin Yvon Fluoromax-4 spectrofluorimeter. Slit width and integration time were kept at 5 nm and 0.2 sec respectively. Tryptophan (Trp) fluorescence was monitored using *λ*_*ex*_ of 295 nm ensuring no contribution from Tyrosine (Tyr) residues. Measurement of fluorescence intensity using excitation and emission at 350 nm and 450 nm respectively indicates formation of AGEs [44]. Excitation at 335 nm and 370 nm correspond to the formation of two AGE products such as pentosidine and malondialdehyde (MDA) [45,46]. The fluorescence intensity values corresponding to different sets (HEWL-glucose/HEWL-fructose/HEWL-ribose) have been corrected with respect to the blank (glucose/fructose/ribose) and thus represent the difference fluorescence intensity in each case.

### Synchronous fluorescence study

Aliquots were withdrawn from HEWL solutions incubated in the presence of glucose, fructose and ribose respectively after an incubation of 31 days at pH 7.4 at 37 °C to examine the synchronous fluorescence. Samples were scanned using a protein concentration of 10 µM between 200 to 600 nm keeping the offset value (Δλ) at 40 nm in Horiba Jobin Yvon Fluoromax 4 spectrofluorimeter [47]. Slit width and integration time were kept at 5 nm and 0.2 sec respectively. The number of band components and respective peak positions were determined from the second derivative spectra.

### Sodium dodecyl sulfate-polyacrylamide gel electrophoresis (SDS-PAGE)

SDS-PAGE was carried out under reducing conditions. Aliquots (100 µM) withdrawn at different times were mixed with sample buffer (2X) containing sodium dodecyl sulfate (SDS) (4 g), bromophenol blue (1%, 4 ml), β-mercaptoethanol (10 ml), glycerol (20 ml) and Tris–HCl [pH 6.8, 1 M, 12.5 ml] (the volume was adjusted to 100 ml with water for the sample buffer). Prior to loading, samples were boiled for 2 min and then applied to a 15% resolving gel and electrophoresis conducted in a Mini Dual vertical electrophoresis unit (Tarson). Gels were stained with Coomassie brilliant blue (SRL, India) and destained using a mixture of CH _3_COOH/MeOH/H _2_O (37.5 ml, 25 ml, 430 ml) with gentle agitation.

### Circular dichroism (CD) measurements

Aliquots withdrawn at definite time intervals were scanned in a Jasco-810 spectrophotometer. Far UV–CD spectra were accumulated between 190 to 240 nm at a scan rate of 50 nm/min keeping the protein concentration at 20 µM using a quartz cuvette having a path length of 0.1 cm. Protein secondary structure contents were estimated using an online server DICHROWEB [48]. Near UV–CD spectra were acquired between 250 to 350 nm at a scan rate of 100 nm/min keeping protein concentration of 100 µM. Sodium phosphate buffer (10 mM) of pH 7.4 was used for dilution in each case.

### SDS-PAGE: detection of glycoprotein using fuchsin staining

SDS-PAGE was performed as mentioned earlier in the previous section. After the completion of the gel run, it was removed and placed in 8%/6% (v/v) AcOH/MeOH solution for gel fixing. The gel was then washed with an oxidizing agent (Sodium periodate in the presence of 2 drops of concentrated H_2_SO_4_) for 15 min. The gel was washed with 3% (v/v) AcOH solution twice for 5 min followed by Fuchsin staining. Destaining was achieved using sodium bisulfite (NaHSO_3_) for 10 min and the gel finally washed with 3% (v/v) AcOH solution to acquire the required staining. Horseradish peroxidase was used as a marker as it is a glycoprotein and develops magenta color in the presence of Fuchsin staining [^4^9, 50].

### Matrix assisted laser desorption ionization-time of flight (MALDI-TOF)

Incubated HEWL solutions were diluted up to a concentration of 100 µM and then mixed with matrix (saturated solution of sinapic acid in 50% (v/v) CH_3_CN/H_2_O solution) in a 1:1 ratio. Samples were then placed on the spot plate, air dried and spectra obtained in a VOYAGER-DE PRO instrument.

### Thioflavin T (ThT) fluorescence

ThT is an amyloid marker dye which intensely fluoresces at ~485 nm upon binding with amyloid fibrils [51,52]. Aliquots from each set were withdrawn at definite interval of days and mixed with ThT to accomplish final protein and dye concentrations of 2 µM and 5 µM respectively. Samples were incubated for 2 min and scanned in a Horiba Jobin Yvon Fluoromax 4 spectrofluorimeter. Excitation and emission maxima were kept at 450 nm and 485 nm respectively. Slit width and integration time were kept at 5 nm and 0.3 sec respectively. Sodium phosphate buffer (10 mM) of pH 7.4 was used for dilution and spectra were corrected with respect to the corresponding blank.

### Fluorescence microscopy

ThT (10 µl of 1 mM) was mixed well with the glycated protein solutions (5 µl) after 30 days and 180 days of incubation to achieve the required staining and placed on a glass slide after covering with a cover slip. Images were obtained using a Leica DM 2500M microscope equipped with a fluorescence attachment. Filter cube no 2 (Leica I3 11513878, BZ: 01) was used for ThT excitation and emission. The images were acquired with a Leica DFC 310 FX camera attached with the microscope. All observations were performed at 10X/0.25 (N PLAN EPI).

### Field emission scanning electron microscopy (FESEM)

Aliquots were withdrawn (2 µl) from each set after an incubation of 60 days at 37 °C at pH 7.4 in the absence and presence of sugars and placed on glass pieces (thoroughly cleaned), air dried and gold coated. Samples were then scanned using a Carl Zeiss field emission electron microscope operating at 5 kV.

### Transmission electron microscopy (TEM)

HEWL solutions incubated in the absence and presence of sugars at 37 °C at pH 7.4 after 180 days of incubation were diluted to a final concentration of 50 µM and placed on TEM grids. Uranyl acetate [1% (w/v)] was used to accomplish the required staining, air dried and scanned in a TECNAI G^2^ 20S-TWIN transmission electron microscope operating at an accelerating voltage of 80 kV.

## Results and Discussion

Glycation results in the structural alteration of proteins. Formation of crossed beta structures in proteins is often associated with glycation and even amyloidal aggregates have been observed as a result of glycation [4,31,32]. Studies have revealed a relationship between the effect of glycation on protein structure, protein aggregation, and fibrillation [31-38]. Recent studies have shown the presence of high levels of AGEs in the brains of patients suffering from neurological disorders and their involvement in amyloid deposition [8,53]. In addition, glycation has been shown to enhance the severity linked with the neurotoxicity of Aβ_1-42_ peptide [12]. Sugars such as glucose, fructose and ribose are common reducing sugars which are well known glycating agents among which the effect of ribose is less studied. In the present article, we have investigated whether prolonged glycation of HEWL can lead to amyloid formation as no such report is available till date, to the best of our knowledge, in case of HEWL. We have used different spectroscopic and microscopic techniques to examine glycation of HEWL and its consequences in aggregation of HEWL in the presence of three reducing sugars such as glucose, fructose and ribose over a prolonged period.

### Fluorescence study: steady state measurements

**Figure 1:**
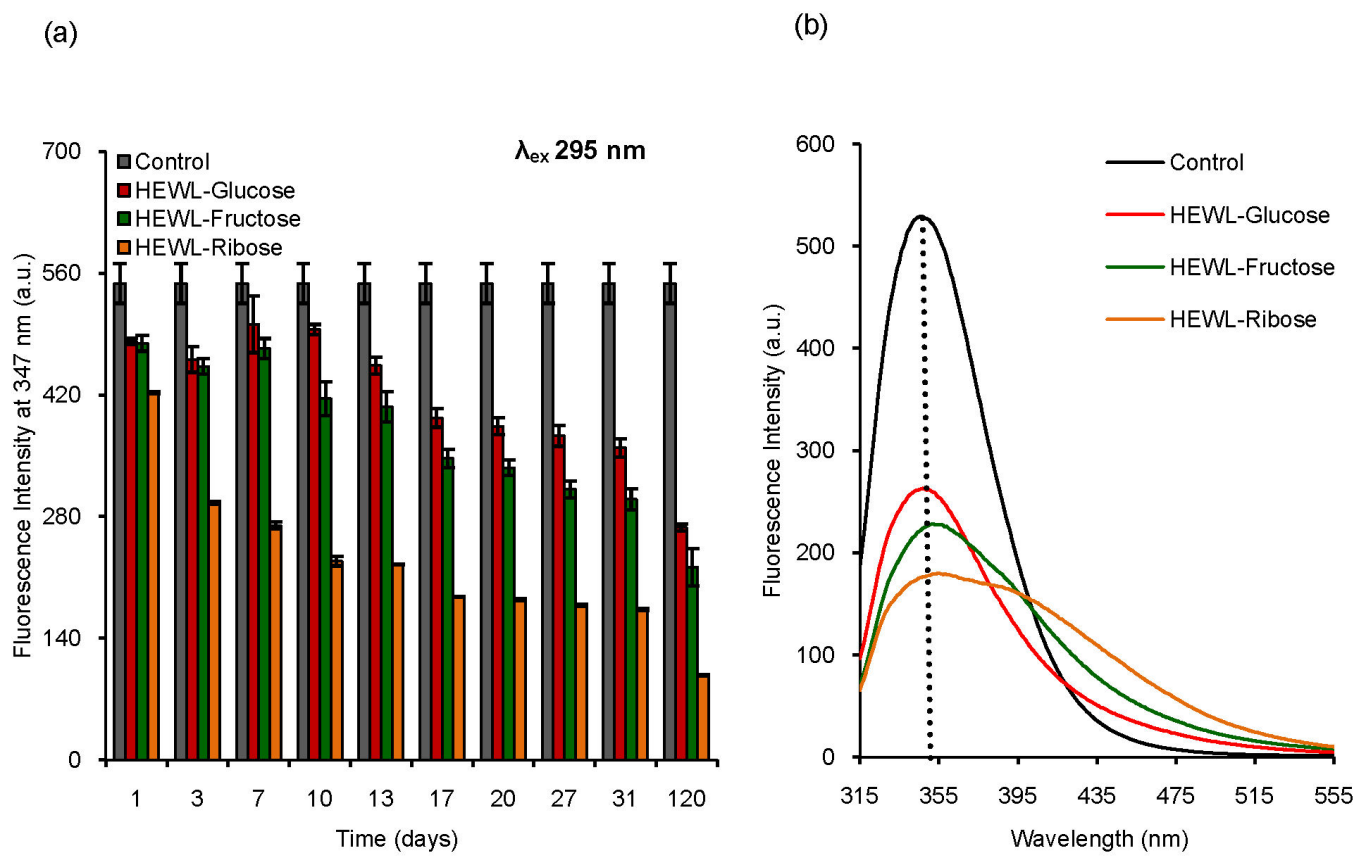
Determination of Trp fluorescence on glycation of HEWL in the presence of different sugars using fluorescence spectroscopy. (a) Histogram represents Trp fluorescence intensity of different HEWL solutions incubated in the presence of glucose, fructose and ribose respectively over a period of 120 days. (b) Representative Trp fluorescence spectra of different HEWL solutions incubated in the presence of glucose, fructose and ribose respectively obtained after an incubation of 31 days. Control represents native HEWL incubated in the absence of sugars at pH 7.4 at 37 °C keeping other conditions similar as that of sets in each case.

**Figure 2:**
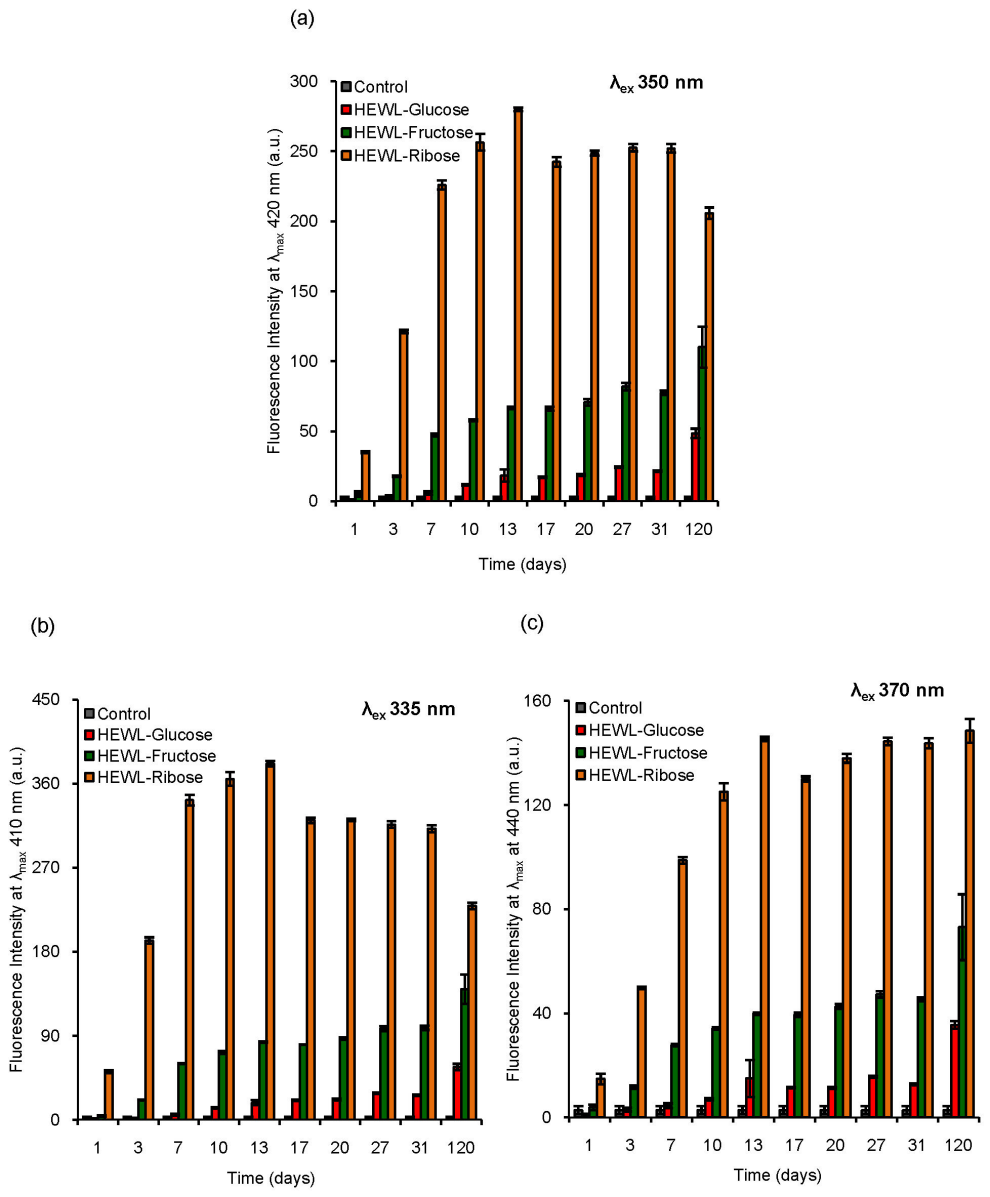
Characterization of different AGE products formed during glycation of HEWL in the presence of different sugars using fluorescence spectroscopy. Histograms represent fluorescence intensity of different HEWL solutions incubated in the presence of glucose, fructose and ribose respectively over a period of 120 days. Formation of different AGE products such as (a) other AGE products (λ_ex_=350 nm), (b) pentosidine (λ_ex_=335 nm) and (c) malondialdehyde (MDA) (λ_ex_=370 nm). [HEWL]=5 µM in each case. Control represents native HEWL incubated in the absence of sugars at pH 7.4 at 37 °C keeping other conditions similar as that of sets in each case.

HEWL was subjected to prolonged glycation in the presence of glucose, fructose and ribose to observe whether glycation could lead to amyloid formation of HEWL. Steady state fluorescence methods were employed to investigate structural changes of HEWL during the incubation period. Formation of AGEs is monitored using excitation and emission at 350 nm and 450 nm respectively [44]. Two different AGEs, pentosidine and malondialdehyde (MDA) formation can be monitored using λ_ex_ values of 335 nm and 370 nm respectively [45,46]. We have measured the fluorescence properties of incubated HEWL solutions using λ_ex_ values of 295 nm, 335 nm, 350 nm and 370 nm respectively [Figure 1 and Figure 2]. We have found a considerable reduction in Trp (λ_ex_=295 nm) fluorescence with increasing incubation period (~120 days) in the presence of each sugar [Figure 1 (a)]. The relative reduction in Trp fluorescence intensity with respect to the control is maximum for ribose (~83% loss) followed by fructose (~60% loss) and glucose (~51% loss). The Trp fluorescence spectra of HEWL solutions after an incubation of 31 days are given in Figure 1 (b). We have noticed that λ_max_ of Trp fluorescence of the control remains the same as would have been expected [Figure 1 (b)]. In the presence of glucose, no prominent shift in λ_max_ was found whereas the presence of fructose and ribose results in a red shift of ~8 nm and ~30 nm respectively [Figure 1 (b)]. This is indicative of an exposure of Trp residues of HEWL to a more polar medium in presence of sugars which is most pronounced in case of ribose [Figure 1 (b)]. Earlier studies have also shown a loss in Trp fluorescence of human serum albumin (HSA) and γ-crystallin due to glycation [54,55]. The red shift in λ_max_ of Trp fluorescence spectrum of γ-crystallin occurs on glycation which has been attributed to exposure of Trp residues to the solvent medium that is similar to the results obtained in this study [55]. We have found that on excitation at 350 nm (indicative of other types of AGEs), incubated HEWL solutions show strong fluorescence in each case (λ_em_=420 nm) [Figure 2 (a)]. HEWL solutions, upon excitation at 335 nm (λ_em_=410 nm) and 370 nm (λ_em_=440 nm) [Figure 2 (b) and (c)] exhibit intense fluorescence which also increases with increasing incubation period. This clearly indicates the formation of two specific AGE products, pentosidine and malondialdehyde (MDA) respectively. In each case, we have noticed that the formation of AGEs reaches a maximum in the presence of ribose implying that ribose plays the most effective role among the sugars used. This is in good agreement with the previously observed fact that the glycating ability of these sugars lies in the order: D-glucose < D-fructose < D-ribose [56]. The higher glycation ability of ribose is attributed to its puckered aldopentose ring structure, which makes it more reactive towards the amino groups in comparison to the other sugars [57,58]. Therefore, it is evident that treatment with sugars such as glucose, fructose and ribose cause prominent structural changes of HEWL along with the formation of AGE products where the effect of ribose is predominant.

### Synchronous fluorescence

**Figure 3:**
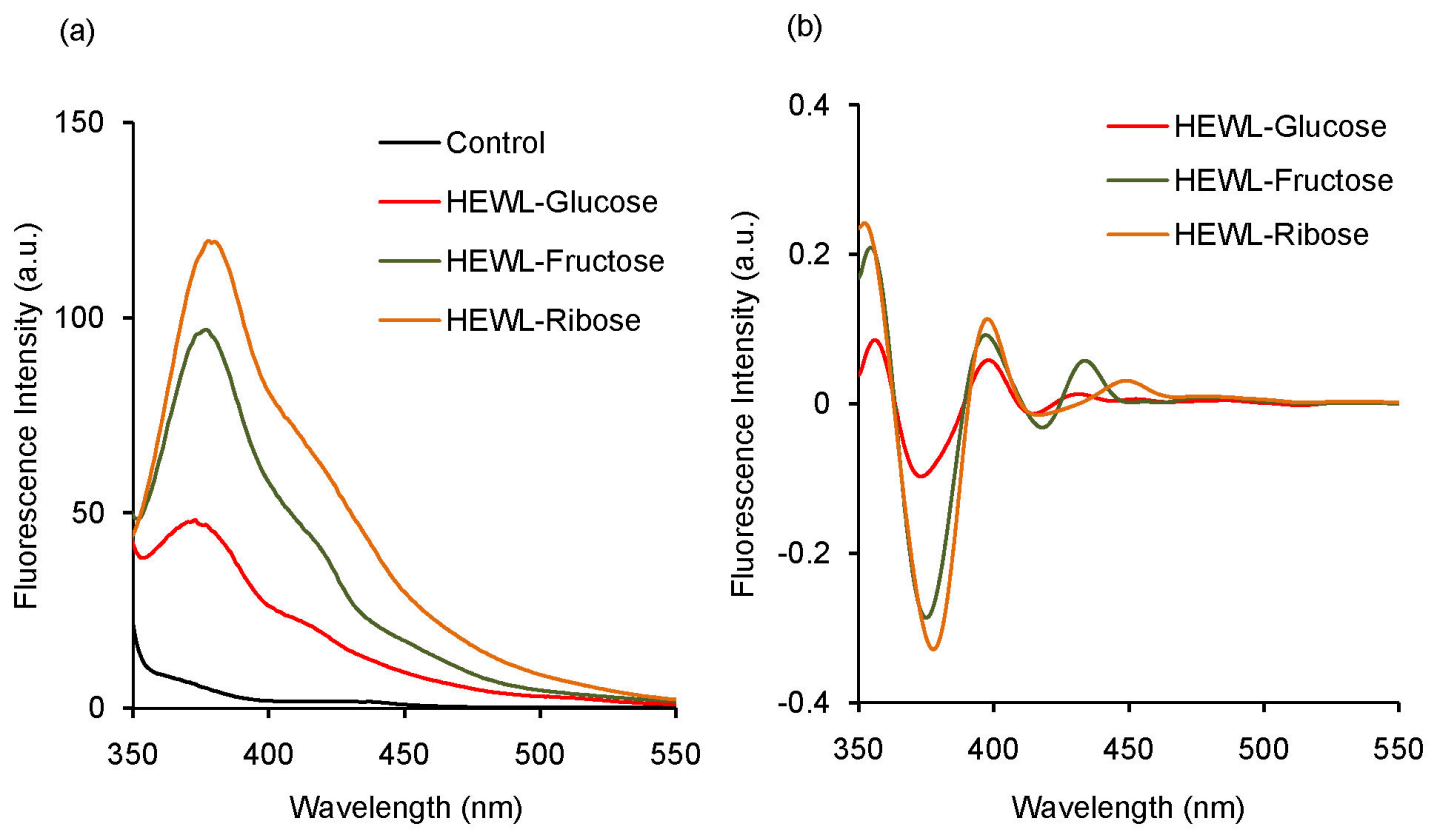
Synchronous fluorescence characteristics of HEWL during glycation in the presence of different sugars. (a) Synchronous fluorescence spectra of control and HEWL solutions treated in the presence of glucose, fructose and ribose respectively after 31 days of incubation at 37 °C at pH 7.4; (b) Second derivative plot of synchronous fluorescence spectra of HEWL solutions incubated in the presence of glucose, fructose and ribose respectively obtained after 31 days of incubation at 37 °C at pH 7.4. Protein concentration=10 µM and Δλ=40 nm in each case.

Synchronous fluorescence studies have been performed to get more detail about the fluorescence property of treated HEWL solutions after 31 days of incubation at 37 °C at pH 7.4. We have noticed that the control does not show any peak in the range from 350 to 550 nm; whereas in the presence of sugars two major peaks are observed [Figure 3 (a)]. The peak near 380 nm is more prominent compared to the other peak near ~415-420 nm in all the cases [Figure 3 (a)]. The maximum intensity of the peak at 380 nm was obtained in the presence of ribose followed by fructose and glucose [Figure 3 (a)]. Our observation is similar to that found earlier in AGE formation of bovine serum albumin (BSA) where ribose was found to be the most potent in generating the 380 nm band [59]. This signifies that the structural changes of proteins are most prominent in the presence of ribose followed by fructose and glucose. To further illustrate this result, we have plotted the second derivative spectra of synchronous fluorescence in each case. We have found two peaks, one at ~375-380 nm and another one at ~415-420 nm respectively [Figure 3 (b)]. Synchronous fluorescence reveals that the effect of ribose is most dominant among the three sugars used which are in accordance with the steady state fluorescence studies.

### Formation of higher oligomers: SDS PAGE

**Figure 4:**
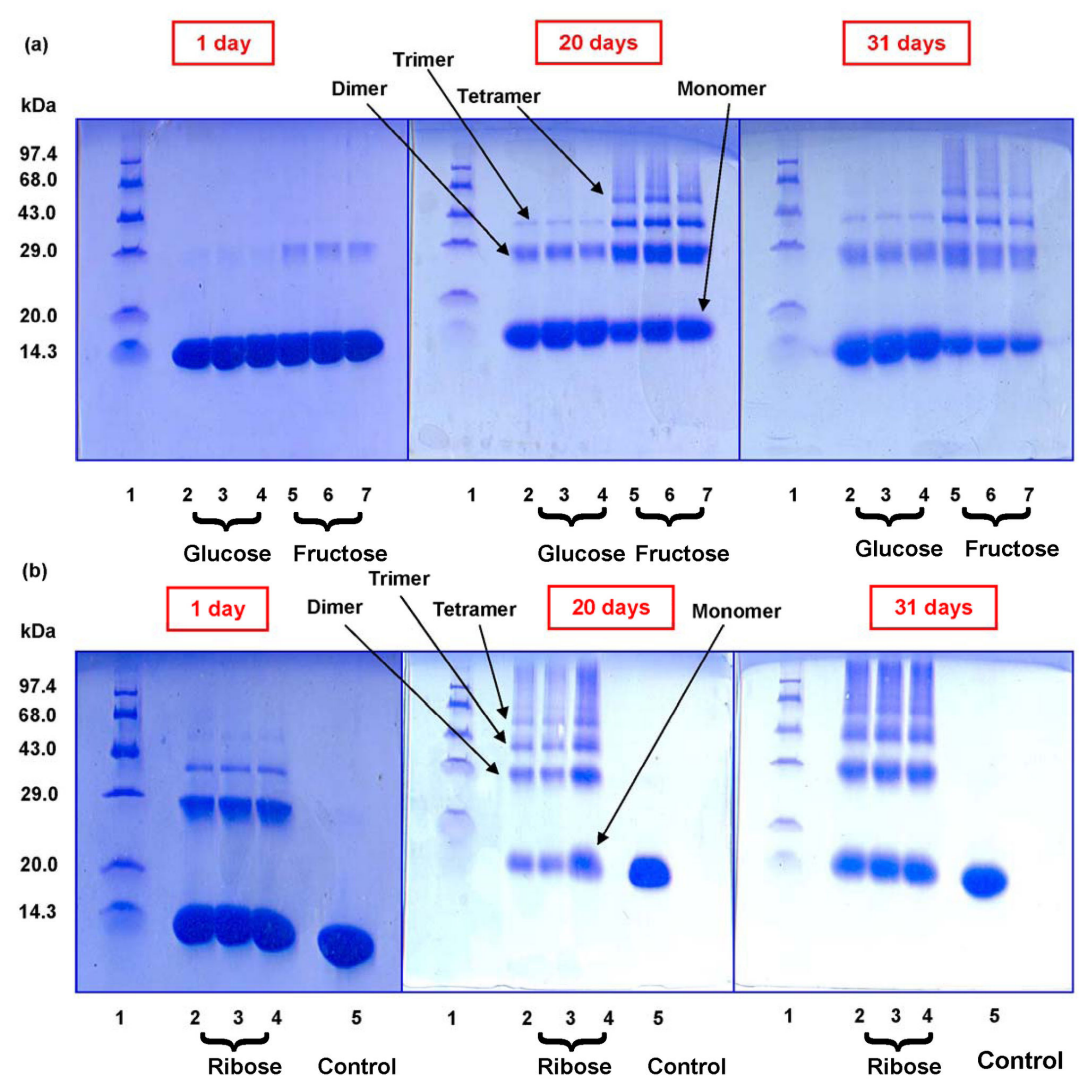
Detection of oligomerization of HEWL during glycation in the presence of glucose, fructose and ribose. Representative SDS polyacrylamide gel electrophoresis of different HEWL solutions obtained after incubation at pH 7.4 at 37 °C in the presence of different sugars at definite intervals of time (1 day, 20 days and 31 days respectively). (a) In each case lane 1: molwt marker; lane 2-4: HEWL-glucose; lane: 5-7: HEWL-fructose respectively; (b) In each case lane 1: molwt marker; lane 2-4: HEWL-ribose; lane: 5: Control (native HEWL incubated at pH 7.4 at 37 °C in the absence of sugars) respectively.

**Figure 5:**
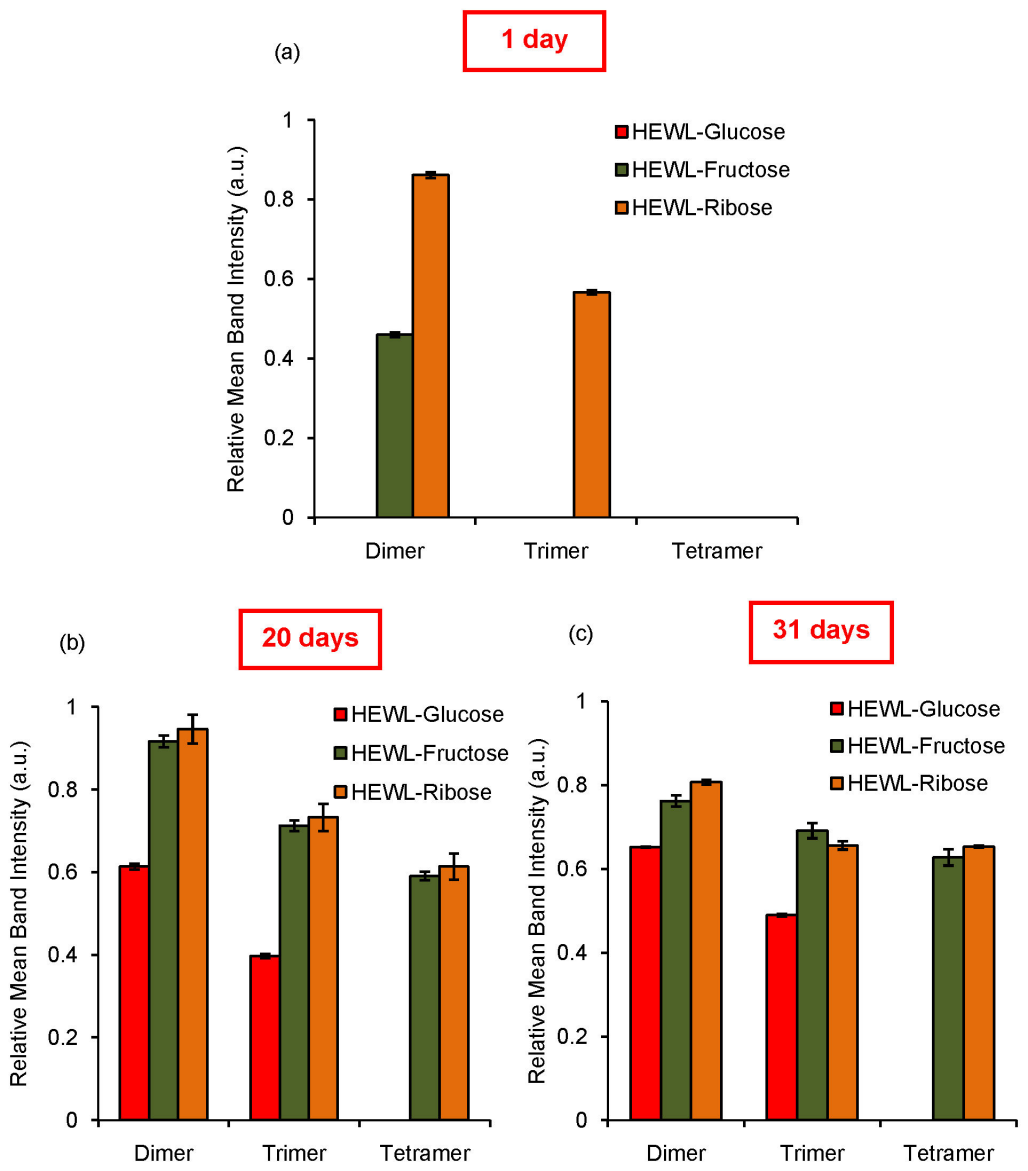
Densitometric analysis of SDS-PAGE. Histograms represent relative mean band intensity of different oligomeric species (dimer, trimer and tetramer) with respect to their corresponding monomer at definite time intervals (a) 1 day, (b) 20 days and (c) 31 days respectively.

To monitor the formation of HEWL aggregates in presence of sugars, SDS-PAGE has been performed under reducing conditions. We have found that after an incubation of 1 day, in the presence of glucose, only monomeric HEWL exists (lanes 2-4) [Figure 4 (a)]. On the other hand, in the presence of fructose, dimeric species are also formed (lane 5-7) [Figure 4 (a)]. Ribose imparts the most noteworthy effect which is clear from SDS-PAGE as trimeric species are formed within a day (lanes 2-4) [Figure 4 (b)]. After 20 days of incubation we have found that even in the presence of glucose, both dimer and trimers were generated (lanes 2-4) [Figure 4 (a)]. Trimer and tetramer formation was also observed in the presence of fructose (lanes 5-7) [Figure 4 (a)] which is most prominent in case of ribose as expected (lanes 2-4) [Figure 4 (b)]. After incubation of 31 days, this becomes more evident as ribose shows formation of oligomeric species in HEWL most effectively followed by fructose and glucose [Figure 4]. This scenario becomes distinctly clear upon quantitation of oligomer formation via densitometric analysis of SDS-PAGE [Figure 5]. We have found that the presence of ribose results in the formation of dimeric species to a higher extent as compared to fructose after 1 day of incubation [Figure 5 (a)]. After 20 days and 31 days of incubation, the formation of higher oligomeric species was found to be maximum in the presence of ribose and minimum in presence of glucose [Figure 5 (b) and (c)]. In each case the relative concentration of oligomers varies in the order: dimer > trimer > tetramer. Therefore, it appears that aggregated cross-linked products of HEWL are indeed formed in the presence of all the three sugars with ribose playing the most potent role. Our observations are similar to those found earlier in the fructosylation reaction of hemoglobin, where the presence of a cross-linked aggregated population was noticed [60].

### Circular dichroism study: conformational aspects of glycation

**Figure 6:**
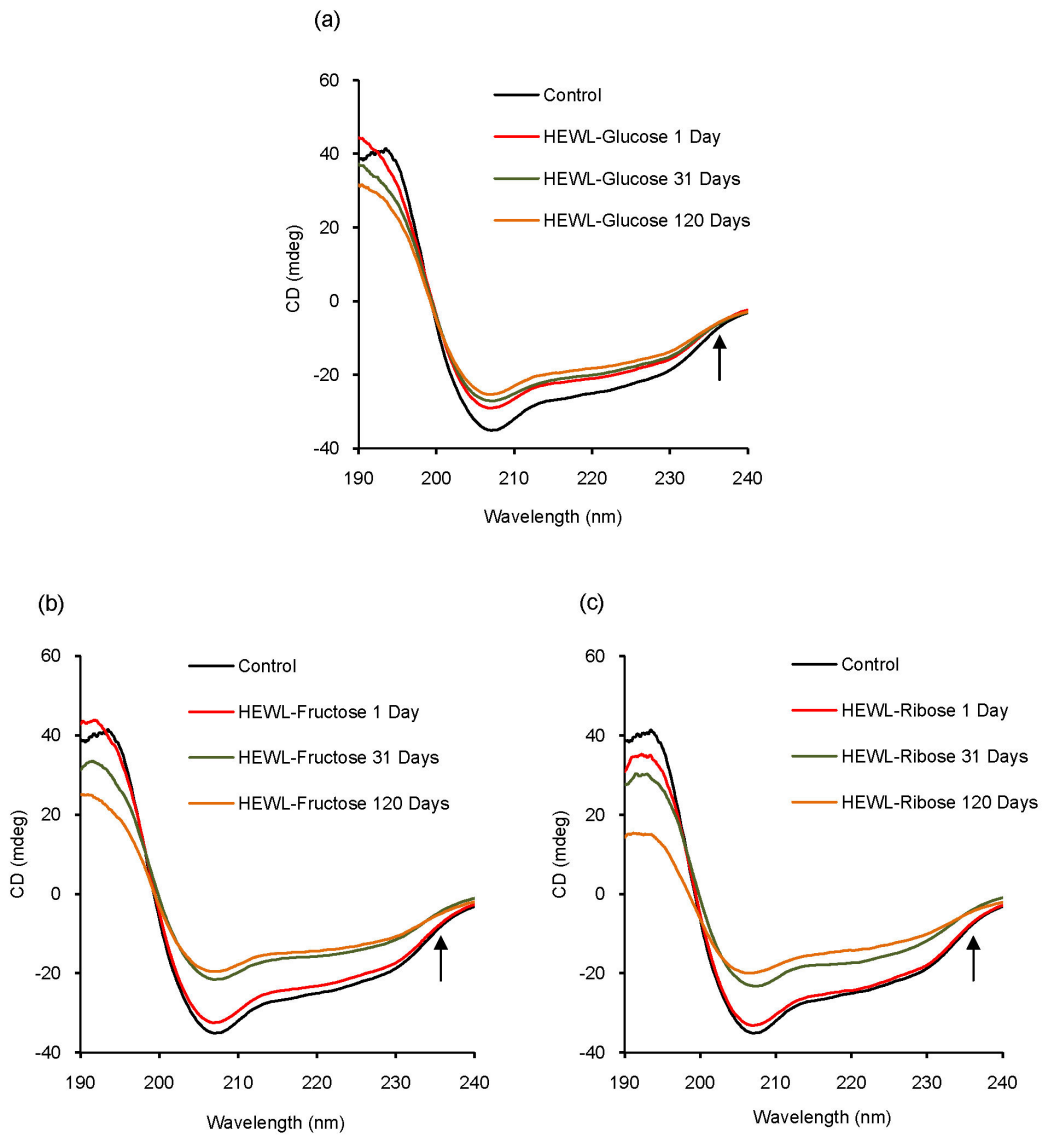
Variation of secondary structural components during glycation of HEWL in the presence of different sugars. Representative far UV-CD spectra of (a) HEWL-glucose, (b) HEWL-fructose and (c) HEWL-ribose solutions respectively obtained after incubation at pH 7.4 at 37 °C at different time intervals. [HEWL]=20 µM in each case.

**Figure 7:**
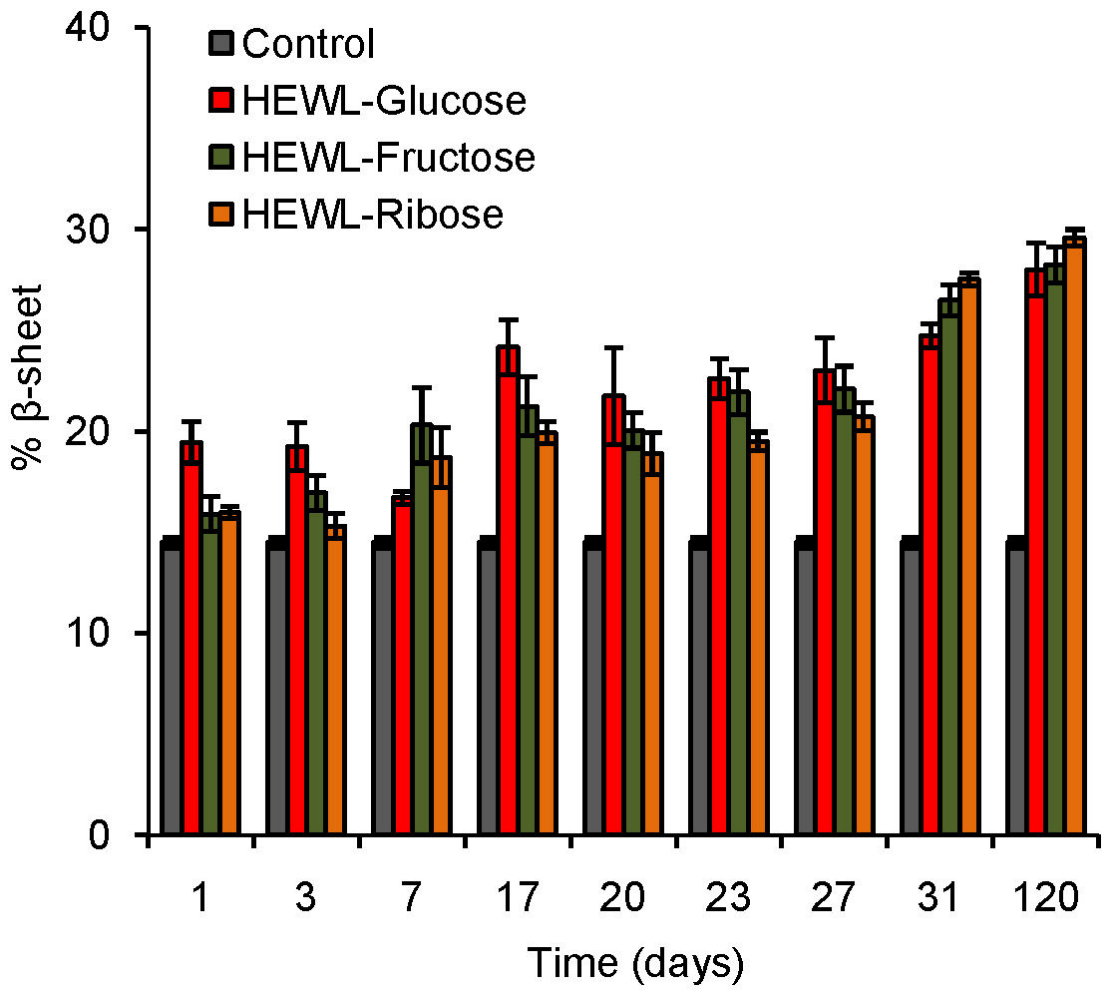
Estimation of β-sheet content of HEWL solutions incubated in the presence of different sugars. Percentage β-sheet content of different HEWL solutions (HEWL-glucose, HEWL-fructose and HEWL-ribose) obtained after incubation at pH 7.4 at 37 °C estimated using online server DICHROWEB at different time intervals.

**Figure 8:**
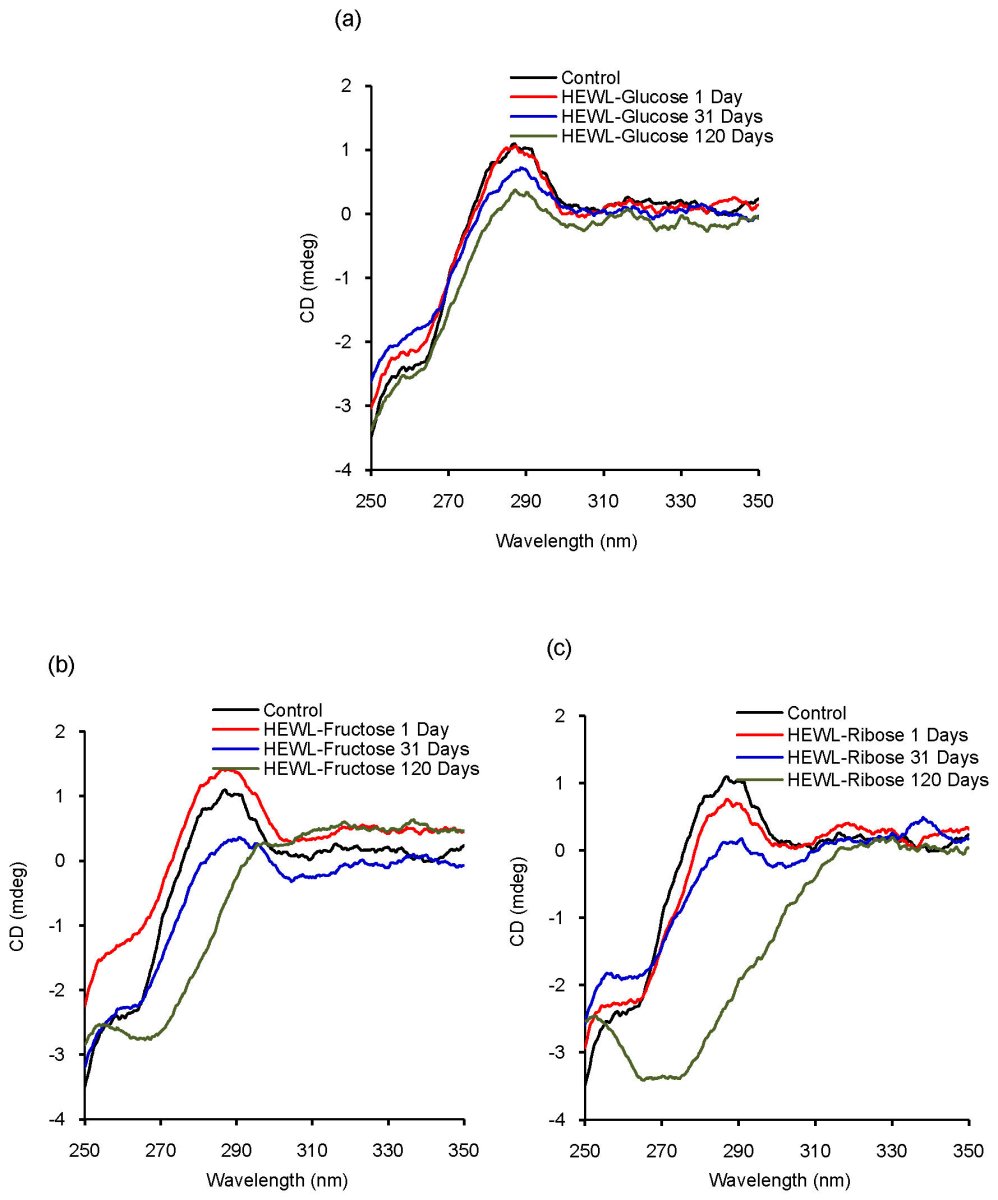
Tertiary structural alterations of HEWL solutions during glycation in the presence of three different sugars. Representative near UV-CD spectra of (a) HEWL-glucose, (b) HEWL-fructose and (c) HEWL-ribose solutions respectively obtained after incubation at pH 7.4 at 37 °C at different time intervals.

Circular dichroism is a useful technique to explore conformational changes in protein structure, especially α-helix to β-sheet transformations [61]. The far UV-CD spectrum of control shows a minimum at 208 nm [Figure 6], which is indicative of α-helical structure [62]. We have noticed that in the presence of glucose, fructose and ribose, the CD mdeg value at 208 nm decreases with increasing incubation period which points toward a loss in helicity [Figure 6]. Lowering in the mdeg value at 208 nm is relatively less in the presence of glucose which is more pronounced in the presence of fructose and ribose. Thus presence of sugars causes prominent secondary structural changes in HEWL (loss in helicity) that is shown to be more effective in the presence of ribose. Quantitative analysis of CD spectra reveals that with increasing incubation period, there is an increase in % β-sheet content of HEWL solutions in presence of all the sugars [Figure 7]. Though increasing trend is more or less maintained throughout the incubation period, we have found slight fluctuation in the % β-sheet content of HEWL solutions in presence of three sugars. Earlier reports have revealed that glycation over a longer period has led to cross-linked beta sheet structure which might be true in this case as well [4]. Near UV-CD spectra of control shows maxima around ~282, ~285 and ~295 nm respectively [Figure 8]. Near UV-CD spectra of HEWL solutions is disrupted to the greatest extent in the presence of ribose followed by fructose and glucose. Thus it becomes evident that maximum perturbation of the tertiary structure of HEWL occurs in the presence of ribose followed by fructose and glucose [Figure 8]. However, in presence of glucose, minimum changes in shape of the spectrum were observed. Thus ribose exhibits most potent role in affecting protein structural changes in comparison to fructose and glucose. This is in accordance with the findings obtained from steady state fluorescence measurements where we have found the maximum red shift in λ_max_ of Trp fluorescence spectrum of HEWL in presence of ribose.

### Detection of glycated proteins: Fuchsin based SDS-PAGE

**Figure 9:**
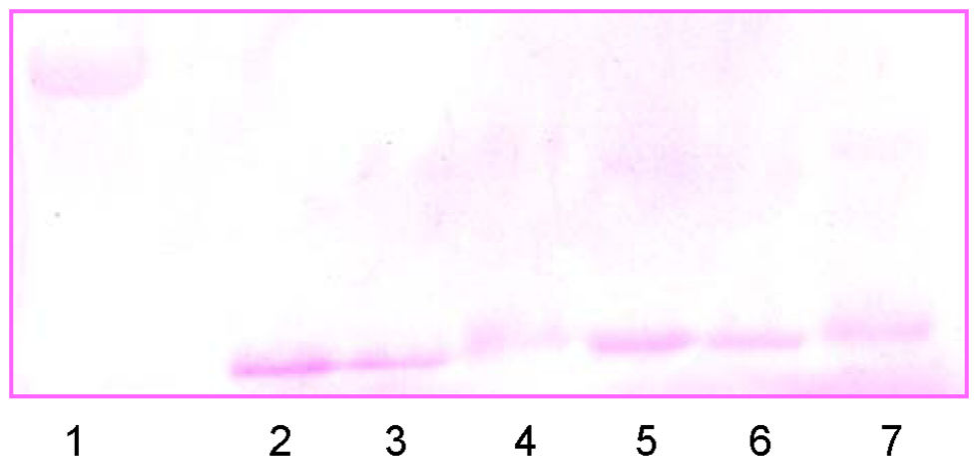
Identification of attachment of sugar moieties to HEWL: Fuchsin based SDS PAGE. Representative SDS polyacrylamide gel electrophoresis of different HEWL solutions obtained after incubation at pH 7.4 at 37 °C for 31 days in the presence of different sugars using Fuchsin staining. lane 1: Horseradish peroxidase; lane 2 and 5: HEWL-glucose; lane 3 and 6: HEWL-fructose; lane 4 and 7: HEWL-ribose.

**Figure 10:**
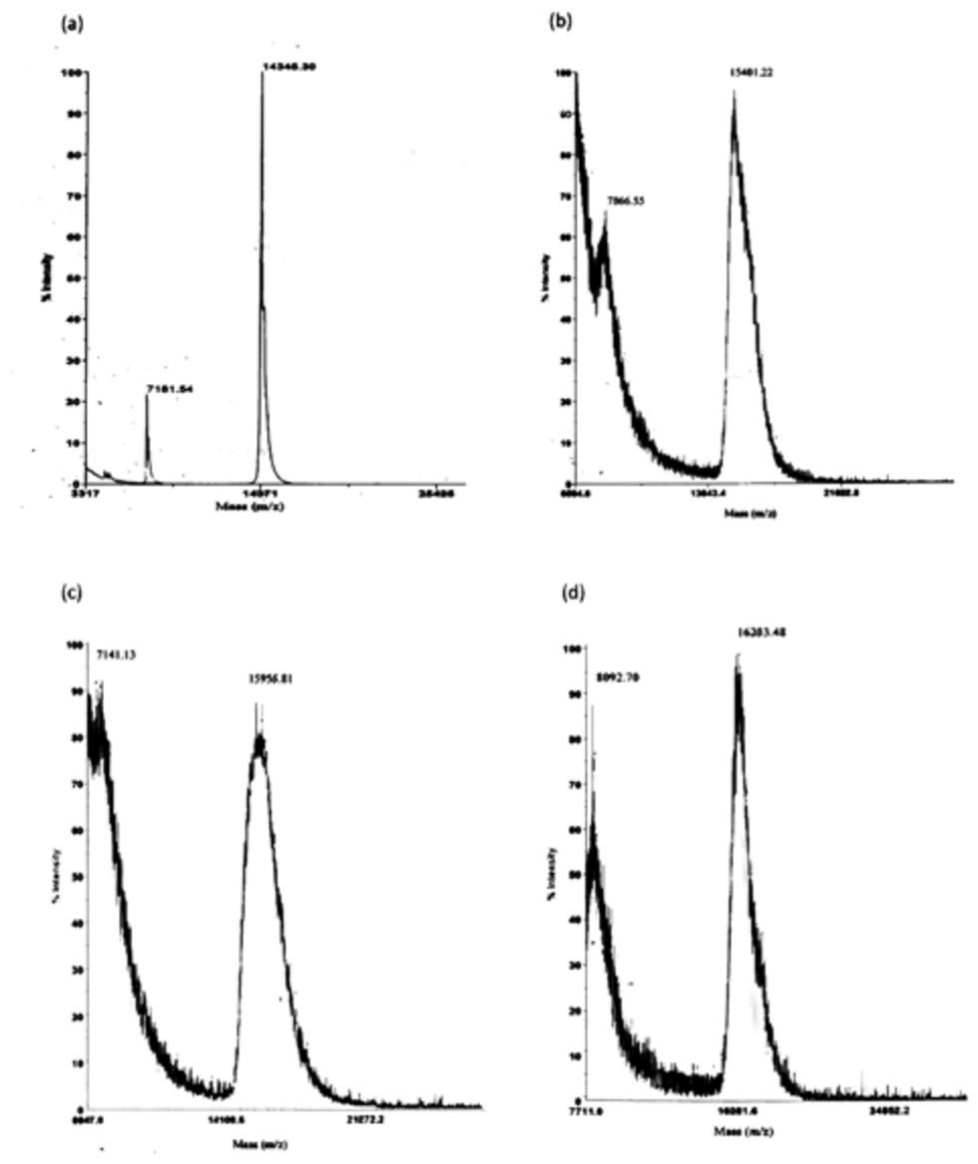
Determination of the mass of glycated HEWL. MALDI TOF spectra of different HEWL solutions obtained after an incubation of 31 days at pH 7.4 at 37 °C (a) Native HEWL (14345.30 Da) (b) HEWL-glucose (15401.22 Da) (c) HEWL-fructose (15955.81 Da) (d) HEWL-ribose (16233.48 Da).

SDS-PAGE has been performed (under reducing conditions) using Fuchsin staining to determine the attachment of the sugar moiety to HEWL. Periodic acid-Schiff base (PAS) reaction is the governing protocol, where periodic acid oxidizes vicinal diol groups to form aldehyde followed by reaction with Schiff reagent to generate a magenta color. This staining is achieved with fuchsin [50]. We have found that horseradish peroxidase, the marker protein is stained in the presence of fuchsin producing a magenta color as would have been expected [Figure 9]. The controls in this experiment are native HEWL and the individual sugars. It was found that they do not develop any color in the Fuchsin based SDS-PAGE assay [Figure S1]. After an incubation of 31 days, HEWL solutions in the presence of glucose (lane 2 and 5), fructose (lane 3 and 6) and ribose (lane 4 and 7) subjected to SDS-PAGE (fuchsin based) develop a magenta color similar to that of horseradish peroxidase [Figure 9]. This clearly indicates the attachment of sugar moiety to HEWL. This further confirms the formation of AGEs in HEWL in the presence of all the sugars which is strongly supported by other studies as well. To further ensure addition of sugar moiety to HEWL, we have performed MALDI-TOF experiments and found that in each case the molecular mass of native HEWL [Figure 10 (a)] increases which corresponds to the attachment of ~6 glucose [Figure 10 (b)], ~9 fructose [Figure 10 (c)] and ~13 ribose [Figure 10 (d)] sugar moieties respectively.

### ThT fluorescence study: nature of the cross-linked aggregates

**Figure 11:**
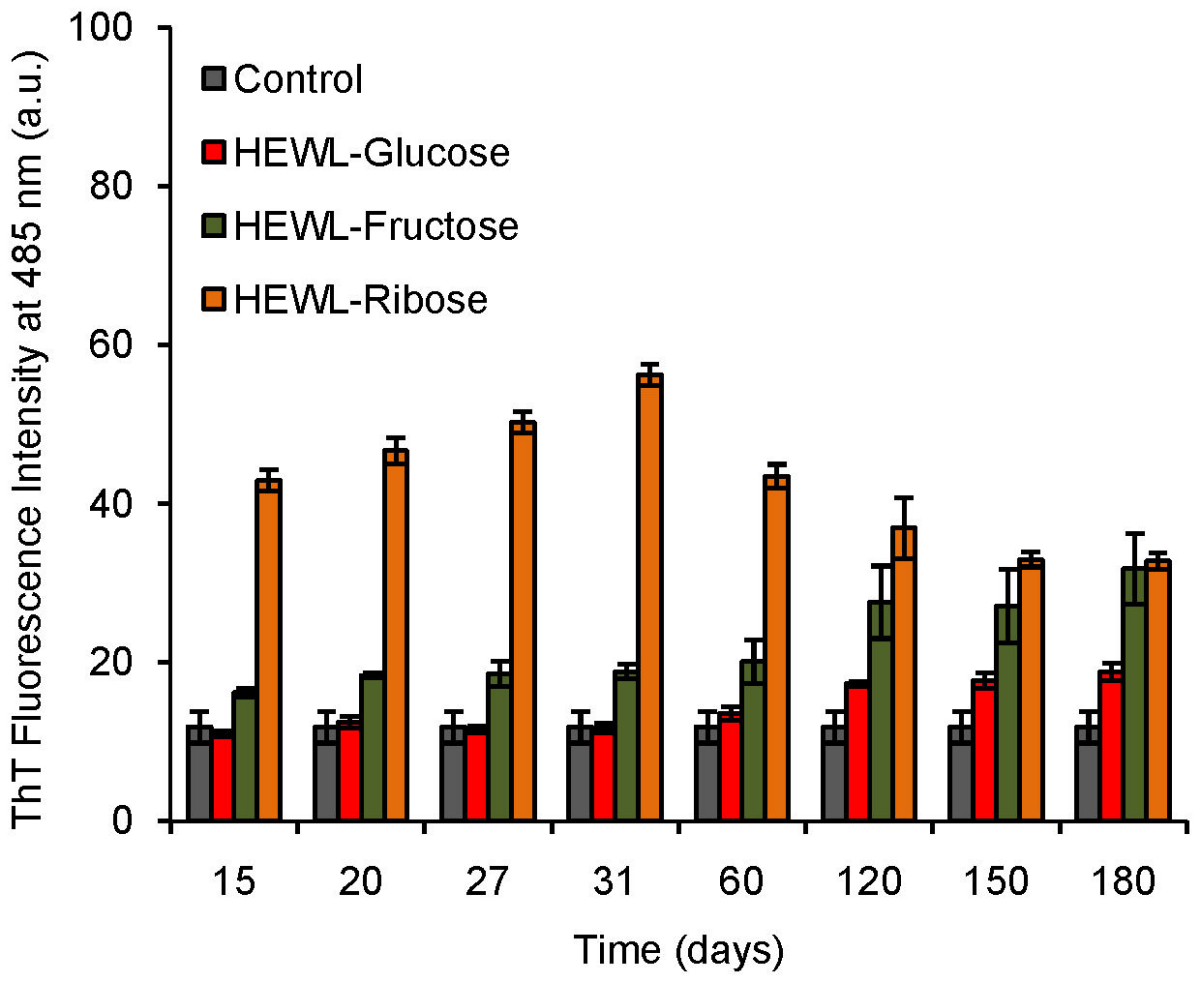
Variation in the ThT intensity during glycation of HEWL in the presence of three different sugars. Histogram represents ThT fluorescence of different HEWL solutions after incubation at pH 7.4 at 37 °C at different time intervals in the presence of different sugars such as glucose, fructose and ribose respectively.

We have found that cross-linked oligomers of HEWL formed during treatment with sugars. We have also noticed that the % β-sheet content of HEWL solutions increases during the incubation period. This has encouraged us to investigate the ThT binding property of these aggregates. ThT is an amyloid specific dye which fluoresces strongly upon binding with amyloid fibrils [51,52]. We have found that up to ~180 days of incubation, there is no significant change in ThT fluorescence for HEWL solutions in the presence of glucose [Figure 11]. HEWL solutions in the presence of fructose show slightly more ThT fluorescence which is ~2.5 times compared to the control solution (native HEWL incubated at pH 7.4 at 37 °C keeping the other conditions similar to that of sets incubated with the individual sugars) [Figure 11]. Ribose treated HEWL solutions show a prominent ThT fluorescence (~5 times compared to the control) for up to ~31 days. After 31 days of incubation we observe a decreasing trend in the ThT intensity (HEWL-ribose solutions) up to ~180 days which then becomes similar to that in the presence of fructose. Thus it appears that HEWL oligomers formed in the presence of ribose possess ThT binding property, other oligomeric species formed in the presence of fructose and glucose do not bind prominently with ThT. We speculate that oligomeric aggregates formed in the presence of glucose and fructose might be amorphous whereas HEWL solutions treated in the presence of ribose might be fibrillar in nature.

### Fluorescence microscopy, FESEM and TEM

**Figure 12:**
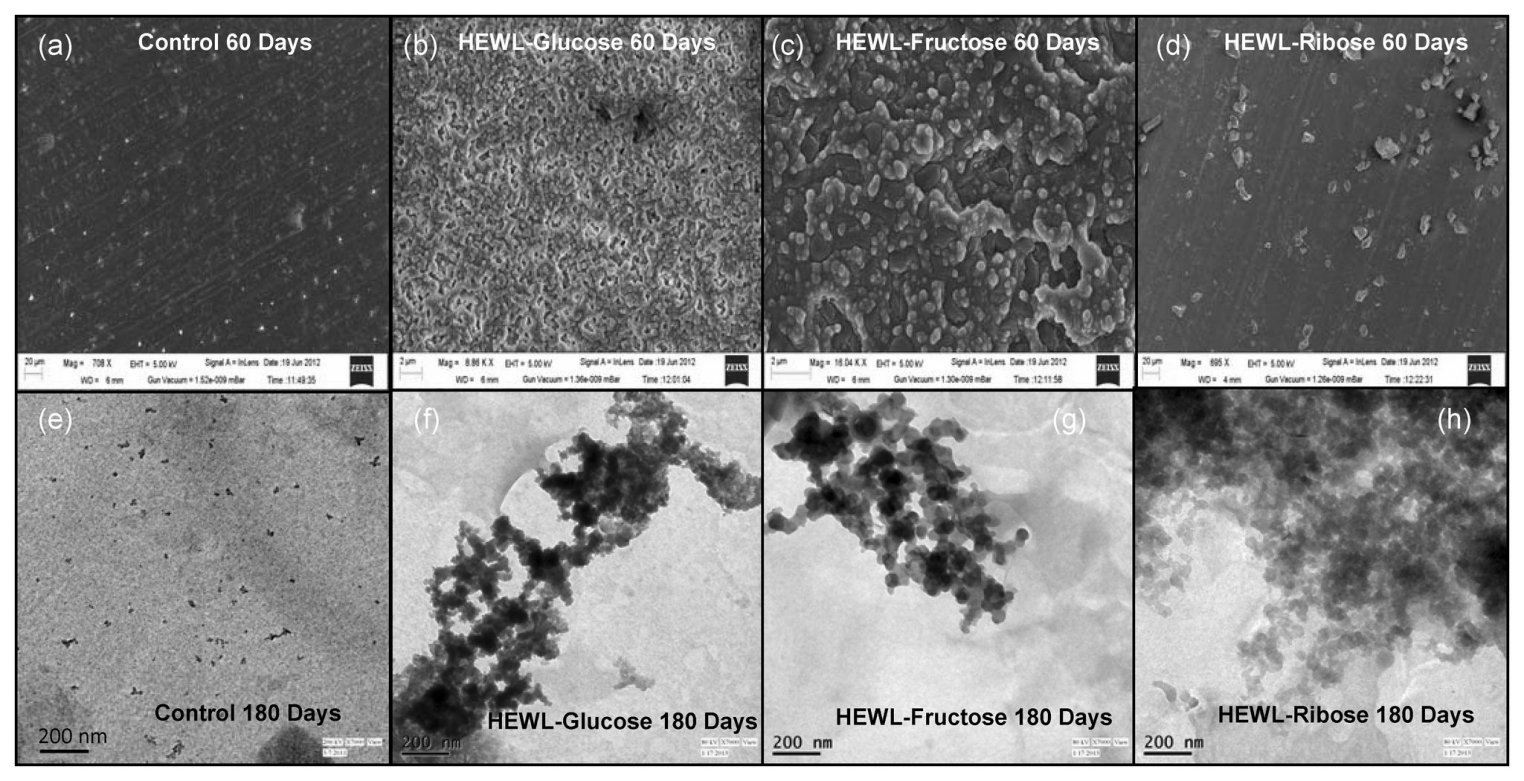
Identification of the nature of the aggregates formed during glycation of HEWL. FESEM (a-d) and TEM images (e-h) of different HEWL solutions obtained after an incubation at pH 7.4 at 37 °C at different time intervals in the presence of different sugars. For FESEM images scale bars represent 2 µm for HEWL-glucose and HEWL-fructose; 20 µm for Control and HEWL-ribose respectively. For TEM images scale bars represent 200 nm.

Microscopic studies were undertaken to analyze the nature of the HEWL aggregates formed. We have found that after 30 days of incubation none of the treated HEWL solutions (in the presence of three sugars) exhibited any prominent fluorescence, not even in the presence of ribose [Figure S2]. Thus HEWL aggregates formed, during the course of incubation in presence of sugars are amorphous in nature. However, after an incubation of 180 days also these treated HEWL solutions do not show any notable change [Figure S2]. Thus it is evident that HEWL does not undergo fibrillation during treatment with sugars. To get a further insight about the morphology of the species formed, we have obtained FESEM and TEM images. We have found that none of them display fibrillar morphology, only globular and amorphous structures are visible in the FESEM images after 60 days of incubation where the control does not show any aggregated morphology [Figure 12 (a-d)]. Our findings are similar to the results obtained earlier where glycation (in presence of D-ribose) results in formation of globular aggregates of α-synuclein which show ThT binding affinity [35]. Furthermore, TEM images clearly show that globular structures are visible in all the cases after 180 days of incubation. Here again the control does not exhibit any aggregated morphology [Figure 12 (e-h)]. Therefore it is clear that extended incubation of HEWL with sugars generates amorphous and globular aggregates. A previous study has shown the formation of nanofibrillar structures of HSA in the presence of glucose, fructose and ribose after prolonged glycation [31]. Apart from fibrillar morphology, globular and amorphous types of aggregates were also visible in this case [31]. However, we have found that glycation of HEWL in the presence of three different sugars does not induce amyloid formation even after ~180 days of incubation. Oligomeric species of amorphous and globular morphology were formed with enhanced β-sheet content. Earlier reports have shown that amorphous aggregates could also be cytotoxic in comparison to the fibrillar morphology [63,64]. Therefore, our study adds fruitful information regarding the morphological diversity of protein aggregates. This will be beneficial in terms of a further understanding of the nature and type of protein aggregates generated during glycation of globular proteins.

## Conclusions

In the present article, we have investigated the effect of glycation on HEWL from the structural point of view and its outcome after a prolonged period. We have found that glycation promotes generation of cross-linked oligomers in HEWL instead of amyloidal aggregates. Ribose was found to be the most effective sugar in facilitating HEWL aggregation. Conformational alteration that is helix to β-sheet transition occurs during glycation of HEWL. Though oligomeric species formed in presence of fructose and mainly ribose, possess ThT binding affinity, they did not show any fibrillar morphology in the microscopic studies. Therefore it appears that prolonged glycation of HEWL results in the formation of cross-linked β-sheet rich oligomers which are amorphous and globular in nature. Our study reflects that glycation causes structural changes of HEWL, generates cross-linked oligomers, but failed to generate fibrillar species. An earlier study has shown glycation induced fibrillation of HSA after an incubation of ~20 weeks [31]. Here we have found that even after ~180 days (~24 weeks) of incubation in the presence of sugars, HEWL does not undergo fibrillation. Ribose has been used as a bioactive component over a longer period. *In vivo* administration of ribose is associated with several risk factors such as hypoglycemia, enhanced insulin levels as well as formation of cross-linked protein aggregates (glycation reaction) which results in cellular dysfunction [65]. A recent study has revealed that ribose is an effective glycating agent compared to glucose both *in vitro* and *in vivo* [58]. Our observations will help in structural analysis of sugar induced damages of HEWL which is a structural homologue of human lysozyme (responsible for systemic amyloidosis disease). Glycation has been found to exert notable effects in relation to neurological disorders. Therefore, our study adds meaningful information regarding glycation related structural alterations of globular proteins and their contribution to amyloid formation. Our study also gives an insight into sugar mediated aggregation of HEWL through the formation of cross-linked oligomers (non amyloidal) and specifically emphasizes the importance of ribose in HEWL glycation.

## Supporting Information

Figure S1 **Fuchsin based SDS-PAGE of Controls.** Lane 1: Horseradish peroxidase (marker); lane 2: Control (native HEWL in the absence of sugars incubated at pH 7.4 at 37 °C keeping the other conditions similar as that of sets); lane 3: glucose; lane 4: fructose; lane 5: ribose.
(TIF)

Figure S2 **Characterization of oligomeric species formed during glycation of HEWL in the presence of different sugars.** Fluorescence microscopic images of different HEWL solutions after incubation at pH 7.4 at 37 °C in the presence of different sugars such as glucose, fructose and ribose respectively at different time intervals (a-d) 30 days, scale bars represent 100 µm; (e-h) 180 days, scale bars represent 20 µm.
(TIF)
