# Supplementary figures and images for: Prolonged Glycation of Hen Egg White Lysozyme Generates Non Amyloidal Structures
Source: PLoS One. 2013 Sep 16;8(9):e74336. doi: 10.1371/journal.pone.0074336 (PMC3774808; doi:10.1371/journal.pone.0074336)

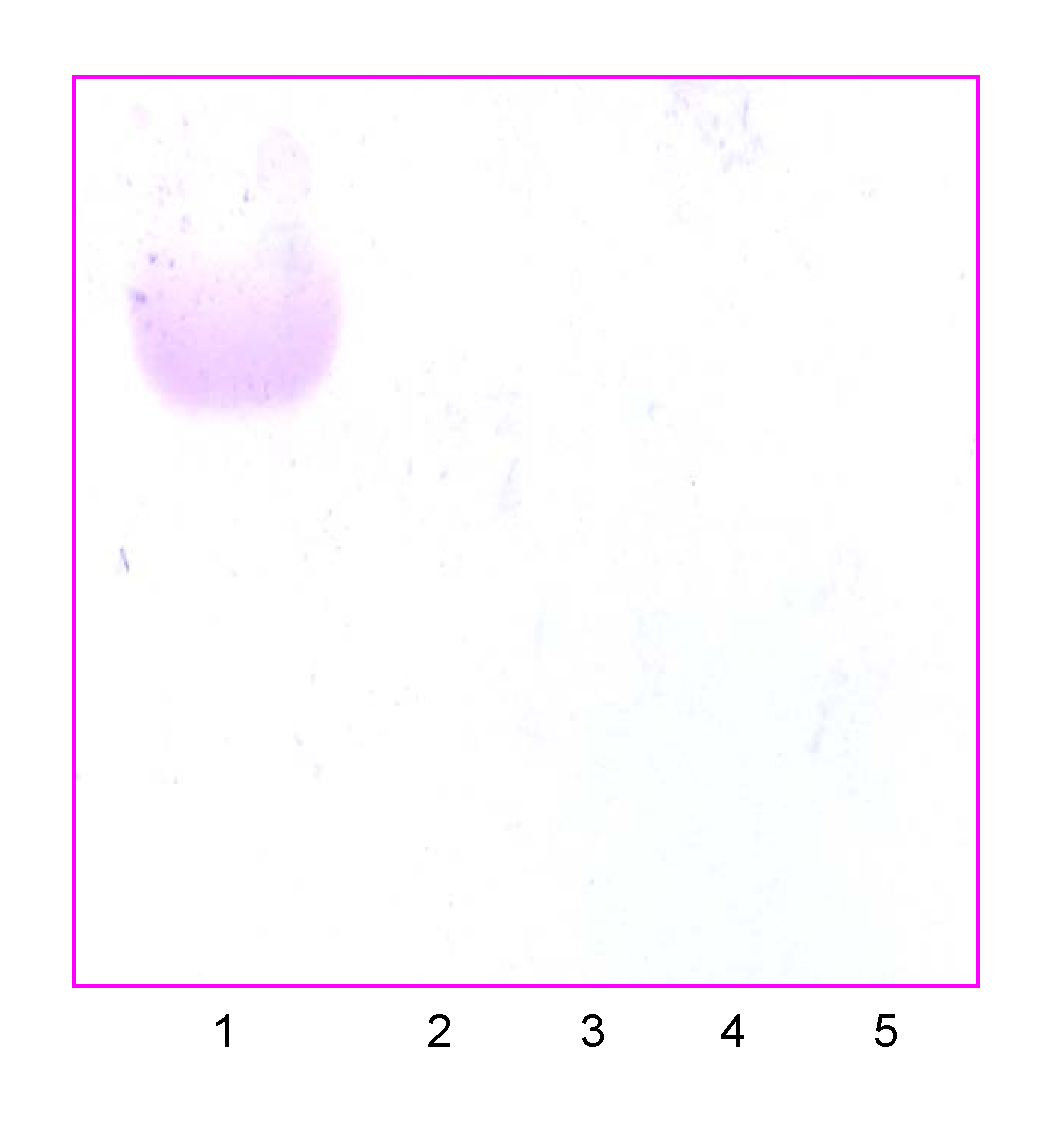

Supplement: Figure S1 — Fuchsin based SDS-PAGE of Controls. Lane 1: Horseradish peroxidase (marker); lane 2: Control (native HEWL in the absence of sugars incubated at pH 7.4 at 37 °C keeping the other conditions similar as that of sets); lane 3: glucose; lane 4: fructose; lane 5: ribose. (TIF) [file pone.0074336.s001.tif]

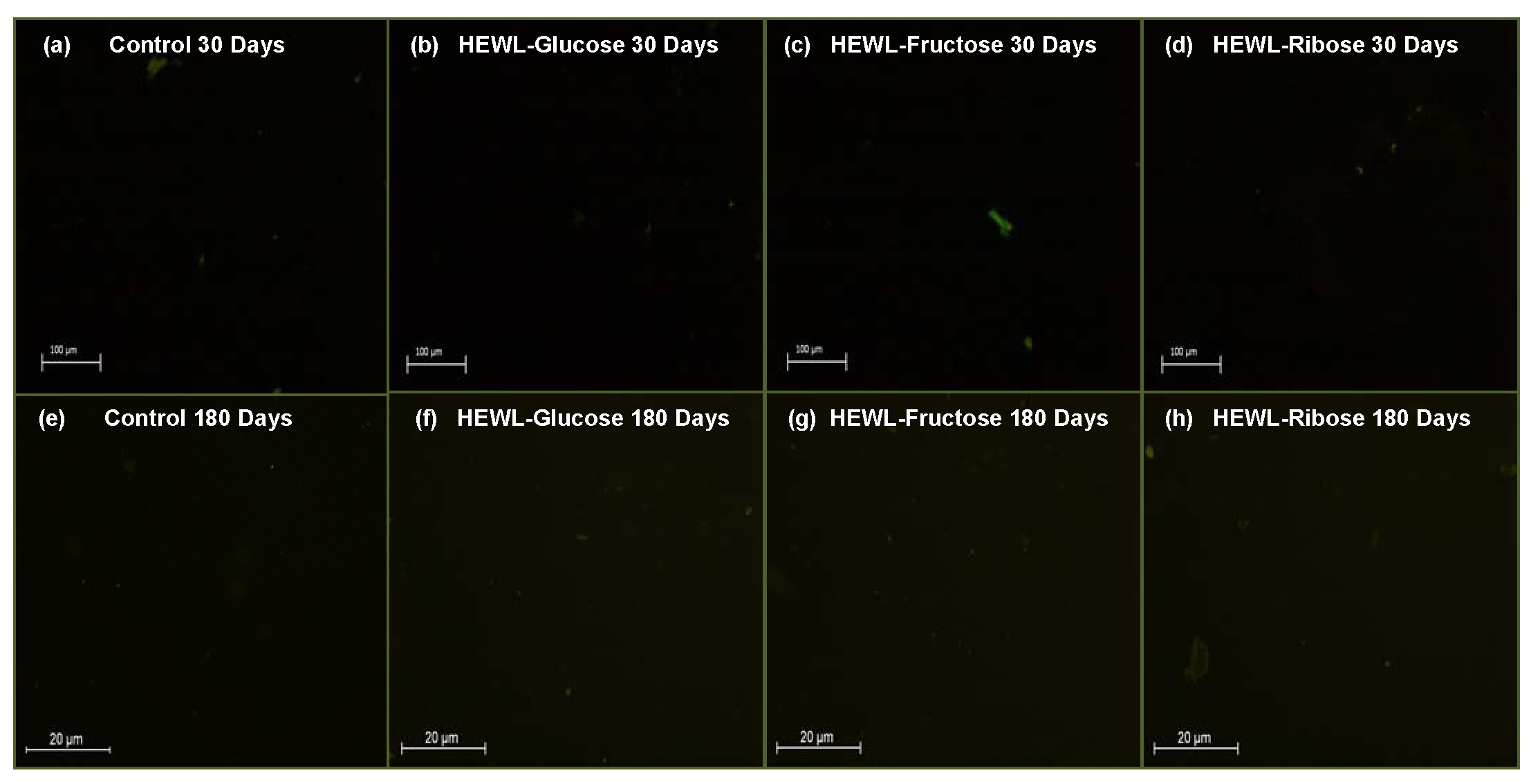

Supplement: Figure S2 — Characterization of oligomeric species formed during glycation of HEWL in the presence of different sugars. Fluorescence microscopic images of different HEWL solutions after incubation at pH 7.4 at 37 °C in the presence of different sugars such as glucose, fructose and ribose respectively at different time intervals (a-d) 30 days, scale bars represent 100 µm; (e-h) 180 days, scale bars represent 20 µm. (TIF) [file pone.0074336.s002.tif]
